# Comparative genomics of *Leishmania donovani* progeny from genetic crosses in two sand fly species and impact on the diversity of diagnostic and vaccine candidates

**DOI:** 10.1371/journal.pntd.0011920

**Published:** 2024-01-31

**Authors:** Jovana Sádlová, Matthew Yeo, David S. Mateus, Jody Phelan, Le Anh Hai, Tapan Bhattacharyya, Stefan Kurtev, Ondrej Sebesta, Jitka Myskova, Veronika Seblova, Björn Andersson, Paola Florez de Sessions, Petr Volf, Michael A. Miles

**Affiliations:** 1 Department of Parasitology, Faculty of Science, Charles University, Prague, Czech Republic; 2 Faculty of Infectious and Tropical Diseases, London School of Hygiene and Tropical Medicine, London United Kingdom; 3 Laboratory of Confocal and Fluorescence Microscopy, Faculty of Science, Charles University, Prague, Czech Republic; 4 Department of Cell and Molecular Biology, Karolinska Institute, Stockholm, Sweden; 5 Genome Institute of Singapore, Biomedical Sciences Institutes, Agency for Science, Technology and Research, Singapore; Universidade Federal de Minas Gerais, BRAZIL

## Abstract

Sand fly transmitted *Leishmania* species are responsible for severe, wide ranging, visceral and cutaneous leishmaniases. Genetic exchange can occur among natural *Leishmania* populations and hybrids can now be produced experimentally, with limitations. Feeding *Phlebotomus orientalis* or *Phlebotomus argentipes* on two strains of *Leishmania donovani* yielded hybrid progeny, selected using double drug resistance and fluorescence markers. Fluorescence activated cell sorting of cultured clones derived from these hybrids indicated diploid progeny. Multilocus sequence typing of the clones showed hybridisation and nuclear heterozygosity, although with inheritance of single haplotypes in a kinetoplastid target. Comparative genomics showed diversity of clonal progeny between single chromosomes, and extraordinary heterozygosity across all 36 chromosomes. Diversity between progeny was seen for the HASPB antigen, which has been noted previously as having implications for design of a therapeutic vaccine. Genomic diversity seen among *Leishmania* strains and hybrid progeny is of great importance in understanding the epidemiology and control of leishmaniasis. As an outcome of this study we strongly recommend that wider biological archives of different *Leishmania* species from endemic regions should be established and made available for comparative genomics. However, in parallel, performance of genetic crosses and genomic comparisons should give fundamental insight into the specificity, diversity and limitations of candidate diagnostics, vaccines and drugs, for targeted control of leishmaniasis.

## Author summary

Sand flies transmit *Leishmania* infections that cause devastating and potentially fatal infectious human diseases. The existence of genetic exchange between *Leishmania* populations has been proved in nature. We used laboratory techniques to create experimental hybrids comparable to those that arise in nature, and analysed the genomes of the resulting offspring. We found vast genetic exchange and diversity across all 36 *Leishmania* chromosomes of the hybrid (diploid) progeny. Genes that are involved in *Leishmania* parasite virulence may recombine into new forms that evade the human immune system. This genetic exchange was also shown to modify and disrupt candidate diagnostic antigens.

## Introduction

An estimated 350 million people worldwide are at risk of leishmaniasis. The disease is prevalent over vast geographical ranges and many countries, notably in eastern Africa, the Indian subcontinent, South America, Central Asia and the Mediterranean region. The *Leishmania donovani* complex (*L*. *donovani* and *L*. *infantum*) causes widespread fatal human visceral leishmaniasis (VL, kala-azar), which is the most devastating form of leishmaniasis, predominantly associated with vulnerable populations, and opportunistic in individuals compromised by HIV co-infection. Without effective treatment symptomatic VL is almost always fatal; around 50,000 to 90,000 cases occur annually [1]. As well as human VL, *L*. *infantum* is responsible for widespread canine visceral leishmaniasis (CVL). Furthermore, *L*. *braziliensis* of the American subgenus *Viannia* causes a severe destructive mucocutaneous leishmaniasis (MCL), with catastrophic invasion and destruction of the nasopharynx. The mechanisms by which *Leishmania* survive in infected asymptomatic carriers and then relapse opportunistically in immunocompromised individuals, are not fully understood. There are no proven human vaccines, and drugs and diagnostics require substantial improvement [2].

The taxonomy of *Leishmania* is complex. There are now four recognized subgenera, *Leishmania*, *Viannia*, *Mundinia* and *Sauroleishmania* [3]. The two subgenera *Leishmania* and *Viannia* include human parasites, each comprised of several species, which have different abilities to survive in sand fly species, and that have distinct distributions of developmental forms in the sand flies that are their associated vectors [4]. Molecular methods have allowed reassessment of *Leishmania* taxonomy, revealing that some named *Leishmania* species are invalid [5]. Historically, all *Leishmania* were considered to be fundamentally clonal. However, evidence accumulated of genetic exchange within and between natural populations of several *Leishmania* species, including those of *Viannia*, such as *L*. *braziliensis* and *L*. *peruviana/guyanensis*, and inbreeding has been detected in natural populations of both *L*. *braziliensis* and *L*. *guyanensis* [6]. Applying multilocus sequence typing (MLST) and microsatellite analysis (MLMT) revealed evidence of sympatric putative parents and hybrid progeny of *L*. *donovani* in an endemic focus of VL in Ethiopia, subsequently confirmed by detailed comparative genomics [7]. Genetic exchange has also been observed among other *Leishmania* populations, for example *Leishmania tropica* of the Middle East [8].

Experimental genetic crosses of *L*. *major* were finally achieved in the sand fly vector, and progeny from geographically disparate sources of *L*. *major* were shown to be viable. The hybrids had genotypes consistent with classical meiosis. However, aneuploidy, recurrent triploidy and loss of heterozygosity (LOH) were also observed [9,10]. More recently, genetic crosses of *L*. *donovani*, *L*. *infantum*, and *L*. *braziliensis* have been achieved *in vitro*, facilitated when the *Leishmania* were exposed to stress and DNA damage, with intraspecific hybridisation, although principally producing tetraploid hybrids [11–13]. Similarly, interspecies crosses between *L*. *donovani* and *L*. *major*, and *L*. *donovani* and *L*. *tropica*, have been achieved *in vitro* [14]. No distinct male or female gametes of *Leishmania* have yet been described, although haploid stages of the trypanosomatid *Trypanosoma brucei* have been discovered in tsetse flies [15].

A range of sand fly species can now be grown in laboratory colonies, enabling great insight into the life cycles and natural genetics of *Leishmania* in the vector. Genetic exchange in *Leishmania* has profound epidemiological importance. It may lead to hybrid vigour (heterosis), with the emergence and spread of strains that are more virulent or are resistant to drugs, with adaptation to new insect vectors, hosts and ecological niches, with implications on the sensitivity and specificity of diagnostic methods and efficacy of vaccine candidates.

Here, we describe experimental genetic crosses of *L*. *donovani* in two sand fly species, *Phlebotomus orientalis* and *Phlebotomus argentipes*, and compare genomics of the parents and the progeny. We also consider the relevance of genetic diversity and genetic exchange on *Leishmania* diagnostic and vaccine candidates.

## Material and methods

### Sand fly colonies and parasites

Laboratory colonies of four sand fly species, *Phlebotomus orientalis* (from Ethiopia), *P*. *argentipes* (India), *Sergentomyia schwetzi* (Ethiopia) and *P*. *duboscqi* (Senegal), were maintained in the insectary of the Charles University in Prague under standard conditions (at 26°C, fed on 50% sucrose,) with a 14 h light/10 h dark photoperiod as described previously [16]. The latter two sand fly species were included as non-permissive vector controls.

*Leishmania donovani* Parent 1 (P1) (MHOM/ET/2010/GR347) expressing enhanced green fluorescence protein (eGFP) and *L*. *donovani* Parent 2 (P2) (MHOM/ET/2010/AM459) expressing red fluorescent protein (dsRFP), both strains originating from Ethiopia, were cultured in M199 medium (Sigma) containing 10% heat-inactivated foetal calf serum (Gibson) supplemented by 1% BME vitamins (Sigma), 2% sterile human urine, 250 μg/ml amikacin (Amikin, Bristol-Myers Squibb), and 150 μg/ml selective antibiotic G 418 (Sigma) and Hygromycin B (Sigma), respectively [4,7]. To obtain amastigote stages, mouse macrophage line J774 was exposed to stationary-phase parasites at a parasite to macrophage ratio 8:1. Both infected and uninfected macrophages were cultured in RPMI medium containing 10% FBS, 100 U/ml of penicillin, 100 μg/ml of streptomycin, 2mM L-glutamine, and 0.05 mM β-mercaptoethanol (all from Sigma) at 37°C with 5% CO_2_.

### Sand fly infections

Amastigote stages were obtained from co-cultivation with J774 cells for 72 h. Non-internalized parasites were removed by washing 3 times with preheated culture medium. Numbers of parasites per macrophages were counted using fluorescent microscopy. Infected macrophages were removed from the cultivation flasks, centrifuged at 200 g for 10 minutes, washed in saline solution and resuspended in heat-inactivated rabbit blood (Bioveta) for sand fly infections at the concentration of 2 × 10^6^ amastigotes ml^-1^ for eGFP parasites and 5 × 10^6^ amastigotes ml^-1^ for dsRFP parasites. Different concentrations were used to account for different growth rates. Sand fly females were co-infected by feeding through a chicken skin membrane on a suspension of both *Leishmania* strains. Engorged females were separated and maintained in standard conditions, as described above. Females were dissected for subsequent analysis on days 2–3 and 7–10 post-blood meal (PBM) (i.e. before defecation of bloodmeal remnants and post defecation, respectively).

### Recovery of hybrid *Leishmania* from sand flies

Live hybrids were recovered by cultivation in double drug selective medium: sand fly females were anesthetized on ice, sterilized in 70% ethanol and washed in sterile saline solution. Dissection was performed under semi-sterile conditions. Dissected midguts were inoculated into wells of microtitration plates containing 50 μl of M199 medium with 10% heat-inactivated fetal calf serum (Gibson), 1% BME vitamins (Sigma), 2% sterile urine, 250 μg/ml amikacin (Amikin, Bristol-Myers Squibb) and fluorocytosin (1.5 mg/ml). The selective antibiotics were both applied after 24 h of cultivation at a concentration of 200 μg/ml in a total volume 100 μl of media. After 7 days live promastigotes were sub-cultured into 1 ml of the medium with double selective antibiotics at a concentration of 150 μg/ml. Presence of the hybrids in cultures were checked by fluorescent microscopy and positive cultures were cryopreserved in liquid nitrogen for further characterisation of recovered parasites.

### FACS analysis

At different time intervals PBM, guts of infected sand flies were dissected into a small volume of saline solution (100 μl) and filtered using 30 μl filters (Partec) into a further 2 ml of saline solution. Putative hybrid cells were sorted with a BD Influx instrument (BD Biosciences) with the laser emitting at 488 nm wavelength for eGFP and 516 nm wavelength for dsRFP and detection of emission at 530/30 (eGFP positive events) and 585/29 nm (dsRFP positive events). The cytometer was calibrated using both a positive control (eGFP and dsRFP transfected lines of *Leishmania*) and a negative control (wild type *Leishmania* strain). Events showing double positive emission were collected into 384 well glass bottom plates with lid (In Vitro Scientific) with 50 μl of saline solution. After complete cell sorting, glass bottom plates with potential hybrid cells were rapidly vortexed and centrifuged (4000 rpm for 1 min) to concentrate cells on the bottom of the plates. Events showing double-positive emission were analysed using the Inverted fluorescence microscope Delta Vision Core (Life Sciences) to detect single cells co-expressing red and green fluorescence.

### Morphometry of hybrid *Leishmania*

*Phlebotomus argentipes* females co-infected with both parental *L*. *donovani* strains were dissected at days 7–9 PBM and the midguts containing parasites were embedded into Vectashield HardSet Antifade Mounting Medium (Vector laboratories, USA) and examined under the Zeiss LSM 880 confocal microscope. Abdominal and thoracic parts of midguts were embedded and evaluated separately. The spectral characteristics of both eGFP and dsRFP tagged cells were examined using a GaAsP spectral detector separately and subsequent on-line unmixing was performed to distinguish between red and green forms in mixed populations. This approach more effectively separated throughput of eGFP and dsRFP signals. Imaging was performed in Nyquist resolution using tile scan function to cover a larger area using a Plan-Apochromat 25x/0.8 oil immersion objective. For high resolution 3D imaging a Plan-Apochromat 63x/1.4 oil immersion objective was used. A 488 nm laser line for eGFP and 561 nm laser line for dsRFP were used in parallel as the excitation sources. Body length, flagellar length and body width of parasites were measured using Image-J software [17]. Three morphological forms were distinguished, according to Sadlova et al [18]: short promastigotes (SP, body length < 14 μm and flagellar length ⩽twice body length), elongated nectomonads (EN, body length ≥ 14 μm) and metacyclic promastigotes (MP, body length < 14 μm and flagellar length >2 times body length). In total, 333 promastigotes were measured.

### Cloning of hybrid *Leishmania* on solid media

Three batches comprising a total of 19 hybrid cultures from 2 sand fly species (*Phlebotomus orientalis* and *P*. *argentipes*) were transferred to the London School of Hygiene & Tropical Medicine (Table 1) and maintained in α-MEM (Sigma) supplemented with 10% inactivated foetal calf serum (FBS) and both selective antibiotics, each at 150 μg/ml and 25°C. For cloning cultures were grown on solid media agar plates of α-MEM, with 3% agar (Sigma), and both antibiotics, adapted from Yeo et al. [19]. Briefly, 10 μl of a 5 x 10^3^ promastigotes/ml dilution of each culture was spread evenly over the surface of the solid medium, plates sealed with parafilm and incubated at 25°C until individual clonal colonies became visible. One to ten colonies were isolated from each plate and inoculated into 2.5 ml of α-MEM, 10% FBS and both selective antibiotics. The cloned isolates were screened for fluorescence by epifluorescence microscopy and highly expressing cultures were selected. To confirm expression of both fluorescent proteins 1 ml of 1 x 10^7^ promastigotes/ml was mixed with 1 ml of 4% paraformaldehyde in phosphate-buffered saline solution (PBS), incubated for 30 mins at room temperature, washed in 1 ml of PBS, centrifuged at 1800 x *g* for 10 mins, resuspended in 200 μl of PBS, and 100 μl of the suspension applied to individual wells in a multiwell polysine-coated slide, which was left to settle for 30 mins at room temperature. Excess fluid was removing by blotting, the slide washed three times in PBS (5 mins) and left to air dry; 10 μl of Vectashield mounting medium incorporating DAPI (Vector Laboratories, USA) was added to each well, covered with a coverslip and sealed with clear nail polish. Samples were examined using a Zeiss confocal laser scanning microscope and colonies containing dual-fluorescent single organisms were thus identified.

**Table 1:** Nineteen hybrid *Leishmania donovani* cultures produced by experimental sand fly infection, and vector origins.

Batch	Sample ID	Vector
1	ARG1	*P*. *argentipes*
1	ARG2	*P*. *argentipes*
1	ARG3 3/4	*P*. *argentipes*
1	ARG3 3/7	*P*. *argentipes*
1	ORI5	*P*. *orientalis*
1	ORI9	*P*. *orientalis*
1	ORI10	*P*. *orientalis*
2	ARG2/2 P3	*P*. *argentipes*
2	ARG2/3 P3	*P*. *argentipes*
2	ARG2/4 P3	*P*. *argentipes*
2	ARG2/5 P3	*P*. *argentipes*
2	ARG3/1 P1	*P*. *argentipes*
2	ORI2/1 P3	*P*. *orientalis*
3	ARG3/2 P1	*P*. *argentipes*
3	ARG3/2 P2	*P*. *argentipes*
3	ARG3/3 P2	*P*. *argentipes*
3	ARG3/4 P2	*P*. *argentipes*
3	ARG3/5 P2	*P*. *argentipes*
3	ARG3/6 P2	*P*. *argentipes*

### FACS analysis of clones

From the selected cultures 1 x 10^7^ parasites were taken at mid-log phase and washed with PBS three times by centrifugation at 1800 x *g* for 10 mins at 4°C, the pellet resuspended in 300 μl PBS, 700 μl of 100% ice-cold methanol added, and homogenised gently by inverting the tube. After 10 mins incubation at 4°C, suspensions were washed three times by centrifugation at 1800 x *g* for 10 mins with PBS at 4°C and resuspended in 10 ml of PBS (giving a final concentration of 1 x 10^6^ cells/ml). Propidium iodide (PI) and RNAse A were added to a final concentration of 10 μg/ml and 1 μg/ml respectively and incubated for 30 mins at 37°C, protected from light. When bound to DNA and excited at 488 nm PI emits red fluorescence, and the fluorescence emitted by individual cells can be measured and compared to infer ploidy. Fluorescence was detected using a BD FACSCalibur flow cytometer (BD Biosciences, USA) with a setting of 10000 events for each sample. Data were gated to exclude clumps and debris and the cell clusters were plotted as area histograms (FlowJo v10.1).

### Genetic analyses of clones

Several genetic analyses were performed on subsets of the 41 cloned hybrids generated at LSHTM. These were: MLST of nuclear and kinetoplastid targets; whole genome sequencing; targeted amplification of the HASPB locus.

### Confirmation of integration and MLST sequencing

DNA was extracted from clonal cultures using DNeasy Blood & Tissue Kit (Qiagen, USA) during the mid-log phase, according to the manufacturer’s protocol. Using Primer-BLAST software [18] appropriate primers were selected for the regions flanking the plasmid vector genomic insertion, with one primer hybridising within the expression cassette and one to a SSU sequence absent from the plasmid (S1 Fig). Integration in all selected transgenic hybrid clones and parental strains was confirmed by amplification and DNA electrophoresis.

For multilocus sequence typing (MLST) three nuclear genes were selected, encoding an hypothetical protein in chromosome 36 (*Ch36-1130)*, a non-coding sequence in chromosome 36 (*Ch36-0350)*, a hypothetical protein in chromosome 28 (*Ch28)*, together with a kinetoplastid gene encoding cytochrome b, all targets containing known SNPs suitable for analysis of genetic exchange [19] (S1 Table). Amplification conditions were optimized with different annealing temperatures and PCR performed in 20 μl reaction mixture containing 1 μl of sample, 2 μl of 10 x NH4 Reaction Buffer (Bioline, UK), 0.8 μl of 50 mM MgCl_2_ (Bioline), 1.6 μl of 2 mM dNTPs (Bioline), 1 μl of 20 pmol/μl forward primer and reverse primer, 0.2 μl of 5U/μl BIOTAQ DNA Polymerase solution (Bioline) and 12.4 μl of ultra-pure water. Amplification conditions were 5 minutes at 95°C, 40 cycles of: 30 seconds at 95°C, 30 seconds at primer/gene specific T_AN_ (S1 Table), 30 seconds at 72°C, and a final elongation step of 5 minutes at 72°C. Products were confirmed by gel electrophoresis with 1.5% m/v agarose in Tris-acetate-EDTA (TAE) buffer containing 0.1 μl/ml of 10000 x GelRed Nucleic Acid Stain (Biotium, USA). The amplified MLST targets were excised from the gel, purified using QIAEX II gel extraction kit (Qiagen) according to the manufacturer’s instructions.

Sequencing reactions were performed in 10 μl of a mixture containing 1 μl of sample, 0.5 μl of BigDye (ThermoFisher Scientific, USA), 1.7 μl of BigDye reaction buffer (ThermoFisher Scientific, USA), 1 μl of 3.2 pmol/μl primer and 5.8 μl of ultra-pure water. Reaction conditions were 1 minute at 96°C for DNA denaturation and 25 cycles of: 10 seconds at 96°C, 5 seconds at 50°C and 4 mins at 60°C, with a rapid thermal ramp of 1°C/second. DNA was then precipitated with ethanol by adding 8 μl of ultra-pure water and 32 μl of 100% ice cold ethanol to each sample, with incubation for 15 mins. The samples were centrifuged at 3000 x *g* for 45 mins at 4°C, supernatant carefully removed, 50 μl of 70% ice cold ethanol added and after a brief vortex, samples were centrifuged at 2000 x *g* for 20 mins at 4°C. The supernatant was again removed, and samples dried at 90°C for 1 min. 10 μl of Hi-Di formamide (ThermoFisher Scientific) was added to each sample and the sequencing was performed on an ABI3730 machine (Applied Biosystems, USA). The forward and reverse sequences for each gene were aligned to create consensus sequences and identify SNPs by comparison between both parental strains and the hybrids. Nucleotide positions displaying allelic heterozygosity (split peaks) were classified according to IUPAC nomenclature. A total of 24 hybrid progeny clones were analysed, 13 derived from 5 *P*. *argentipes* and 11 from 5 *P*. *orientalis*, together with clones representing the two parents (Table 2).

**Table 2:** Hybrid and parental clones used in MLST analyses.

Sample number	Sample ID	Vector
1 parental	AM459.1	-
2 parental	AM459.2	-
3	ARG 1 C1	*P*. *argentipes*
4	ARG 1 C2	*P*. *argentipes*
5	ARG 1 C3	*P*. *argentipes*
6	ARG 4/1 C1	*P*. *argentipes*
7	ARG 4/1 C2	*P*. *argentipes*
8	ARG 4/1 C3	*P*. *argentipes*
9	ARG 4/2 C2	*P*. *argentipes*
10	ARG 4/2 C3	*P*. *argentipes*
11	ARG 4/2 C4	*P*. *argentipes*
12	ARG 4/3 C1	*P*. *argentipes*
13	ARG 4/3 C2	*P*. *argentipes*
14	ARG 4/5 C1	*P*. *argentipes*
15	ARG 4/5 C2	*P*. *argentipes*
16	ORI 4/1 C1	*P*. *orientalis*
17	ORI 4/1 C2	*P*. *orientalis*
18	ORI 4/3 C1	*P*. *orientalis*
19	ORI 4/3 C2	*P*. *orientalis*
20	ORI 4/5 C1	*P*. *orientalis*
21	ORI 4/7 C1	*P*. *orientalis*
22	ORI 9 C1	*P*. *orientalis*
23	ORI 9 C2	*P*. *orientalis*
24	ORI 9 C3	*P*. *orientalis*
25	ORI 9 C6	*P*. *orientalis*
26	ORI 9 C7	*P*. *orientalis*

### Comparative genomics of parents and hybrid progeny

Whole genomes of both parents and cloned progeny were sequenced at the Karolinska Institute (Sweden) and/or at the Genome Institute of Singapore, using a combination of Illumina and PacBio. A reference assembly was generated for the PacBio sequenced parental strain and was scaffolded using the companion annotation server [20]. Fastq data for parental and progeny strains were aligned to the PacBio reference using bwa (v0.7.17) [21] and processed using samtools (v1.12) [22]. Small variants were called using gatk HaplotypeCaller (v4.1.4.1) [23]. Parent-specific SNPs were found by comparing frequencies of alternate alleles in both parent strains, selecting those that were homozygous for different alleles. Frequencies of parent-specific SNPs were then extracted from progeny and were used to construct bottle brush plots using R to detect recombination. S2 Table lists the accession numbers on the European Nucleotide Archive of the genome sequences from one of the parental and 26 of the hybrid clones.

### HASPB diversity

For HASBP, which is a component of the rK28 antigen used in VL diagnosis and of a therapeutic vaccine under trial [24], two primers (LdonHASPBfor 5’- CATAAAACCACTGAGGC-3’ and LdonHASPBrev 5’-ATCTTCGTTCTTCTCCTG-3’) were designed to amplify the repeated regions in the HASPB1 and HASPB2 genes of three parental samples and 7 hybrid clones, as described above but using annealing temperature of 52°C, and 0.4 μM dNTPs. Each band separated by electrophoresis on 1% agarose gels was excised and purified using QIAquick Gel Extraction Kit, according to manufacturer’s instruction, and BigDye reactions conducted with the same amplification primers. Consensus nucleotide sequences were aligned with *L*. *donovani* reference genome and translated into amino acid sequence, using Bioedit and Chromas software.

### Diversity of diagnostic and vaccine candidates

A wider selection of published proposed *L*. *donovani* vaccine and diagnostic candidates was made to compare the diversity between parents and among progeny. Criteria for selection of targets were: high antigenicity; involvement in surface signalling or vital *Leishmania* metabolism; sequence data or nomenclature that allowed access to such data in the public domain. The gene sequences were extracted from alignments with NCBI database sequences. The SNP-containing sequences were translated to amino acids and a table created with all SNPs classified as either synonymous (no amino acid change), missense (amino acid substitution present) or stop-codon inducing. Finally, a list was created of targets observed to have amino acid sequence diversity (missense and stop codon inducing mutations) impact on diagnostic or vaccine candidates.

## Results

### Detection and recovery of hybrids

Two methods were used to detect primary hybrid *Leishmania* from experimental infection—flow cytometry and cultivation of sand fly guts in double-selective media. Both methods revealed presence of hybrid cells, although only in late stage infections (days 7–9 PBM) from *P*. *orientalis* and *P*. *argentipes*, both of these sand fly species being the proven natural vectors of *L*. *donovani*. Early stage infections (days 2–3 PBM) did not yield any hybrids in all four sand fly species (Table 3 and 4); bacterial or fungal infections were higher in these early stage samples (Table 4). Flow cytometry gave positive results in 2 out of 3 experiments with late stage infections in *P*. *orientalis* and 2 out of 4 experiments with late stage infections in *P*. *argentipes*, although numbers of hybrid cells detected by flow cytometry were very low (Table 3).

**Table 3:** Flow cytometry analysis detecting both red and green fluorescence by confocal microscopy.

Sand fly species	Day post bloodmeal	No. of experiments		No. of dissected sand flies		No. of cells with dual fluorescence	
**Early stage infections**							
*P*. *orientalis*	2	1		118		0	
*P*. *argentipes*	2	4		215		0	
*S*. *schwetzi*	2	2		127		0	
*P*. *duboscqi*	2	2		131		0	
**Late stage infections**							
*P*. *orientalis*	7	3		100		5	
*P*. *argentipes*	7–9	4		219		3	

Footnote: *S*. *schwetzi* & *P*. *duboscqi* were included to provide confirmation that progeny were not achieved in these non-permissive vectors.

**Table 4:** Cultivation of guts from sand flies co-infected with both parental strains of *L*. *donovani* then grown on double drug selective media.

Sand fly species	Day post bloodmeal	No. of experiments	No. of dissected sand flies	Bacterial or fungal contamination	No. of positive / recovered cultures (%)
**Early stage infections**					
*P*. *orientalis*	2	3	47	0	0/47
*P*. *argentipes*	2	4	66	1	0/65
*S*. *schwetzi*	2–3	3	69	17	0/52
*P*. *duboscqi*	2–3	4	101	9	0/92
**Late stage infections**					
*P*. *orientalis*	7–9	3	89	0	14/89 (16)
*P*. *argentipes*	7–10	7	211	1	20/210 (10)

Footnote: *S*. *schwetzi* & *P*. *duboscqi* were included to provide confirmation that progeny were not achieved in these non-permissive vectors.

Recovery of hybrids in double drug selective media was successful with all 3 late stage infections of *P*. *orientalis* and 4 of 7 late stage infections of *P*. *argentipes*. Non-hybrid cells usually survived for up to 1 week. Stably growing cultures were obtained in 16% and 10% of isolates from *P*. *orientalis* and *P*. *argentipes*, respectively (Table 4). All these selected cultures showed both red and green fluorescence by fluorescence microscopy.

### Morphology of hybrid *Leishmania* in sand flies

**Fig 1:**
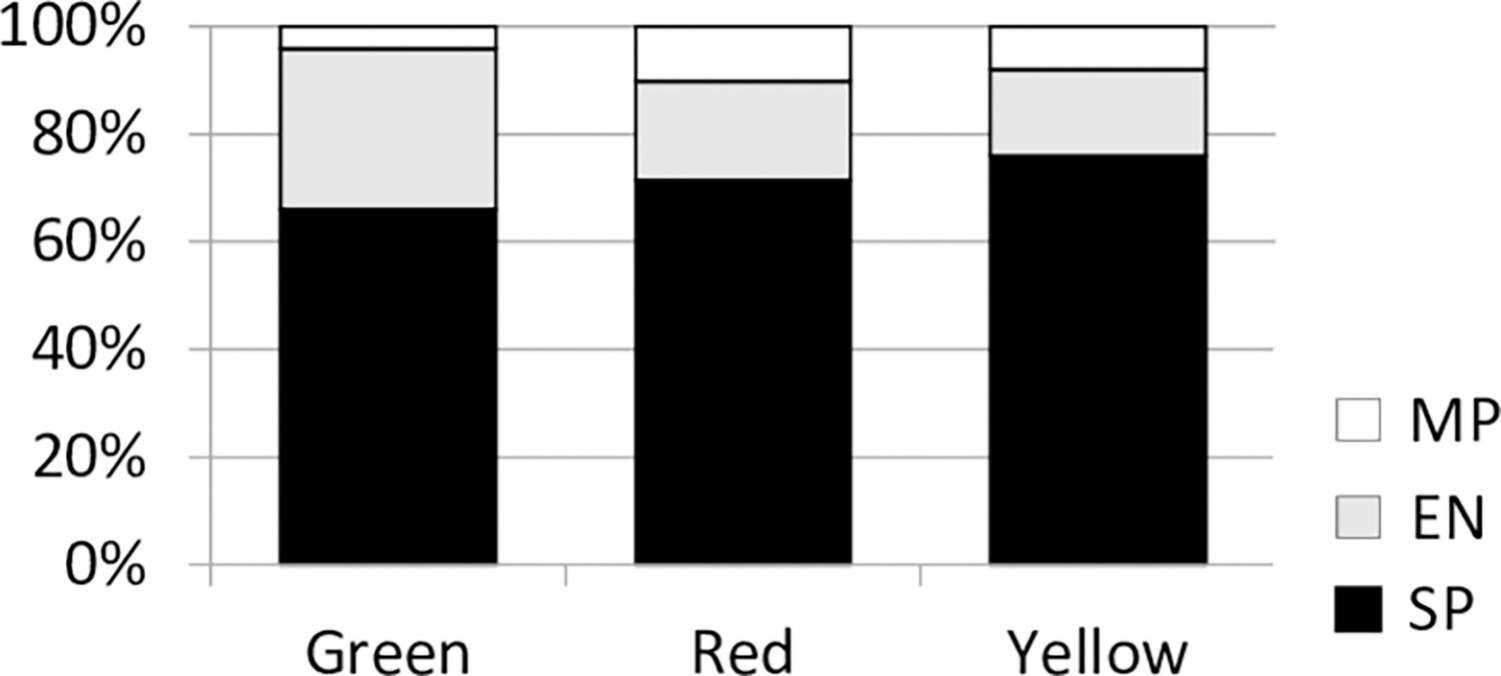
Proportions of three morphological *Leishmania* forms in guts of *P*. *argentipes* at days 7–9 PBM. SP, short promastigotes, EN, elongated nectomonads, MP, metacyclic promastigotes, and potentially hybrid yellow short promastigotes.

Morphology of *L*. *donovani* was evaluated at days 7–9 PBM with gut smears of *P*. *argentipes* co-infected with both parental strains. Hybrid *Leishmania* were present in both abdominal and thoracic parts of the guts. Representation of three morphological forms among parental and hybrid cells bordered on statistical significance (Chi-square = 9.066, d.f. = 4, *P* = 0.059). Elongated nectomonads (EN) were more frequent in the GR347 green parental strain than in the AM459 red strain and hybrids (Fig 1).

### Hybrid clones express both fluorescence proteins and are principally diploid

**Fig 2:**
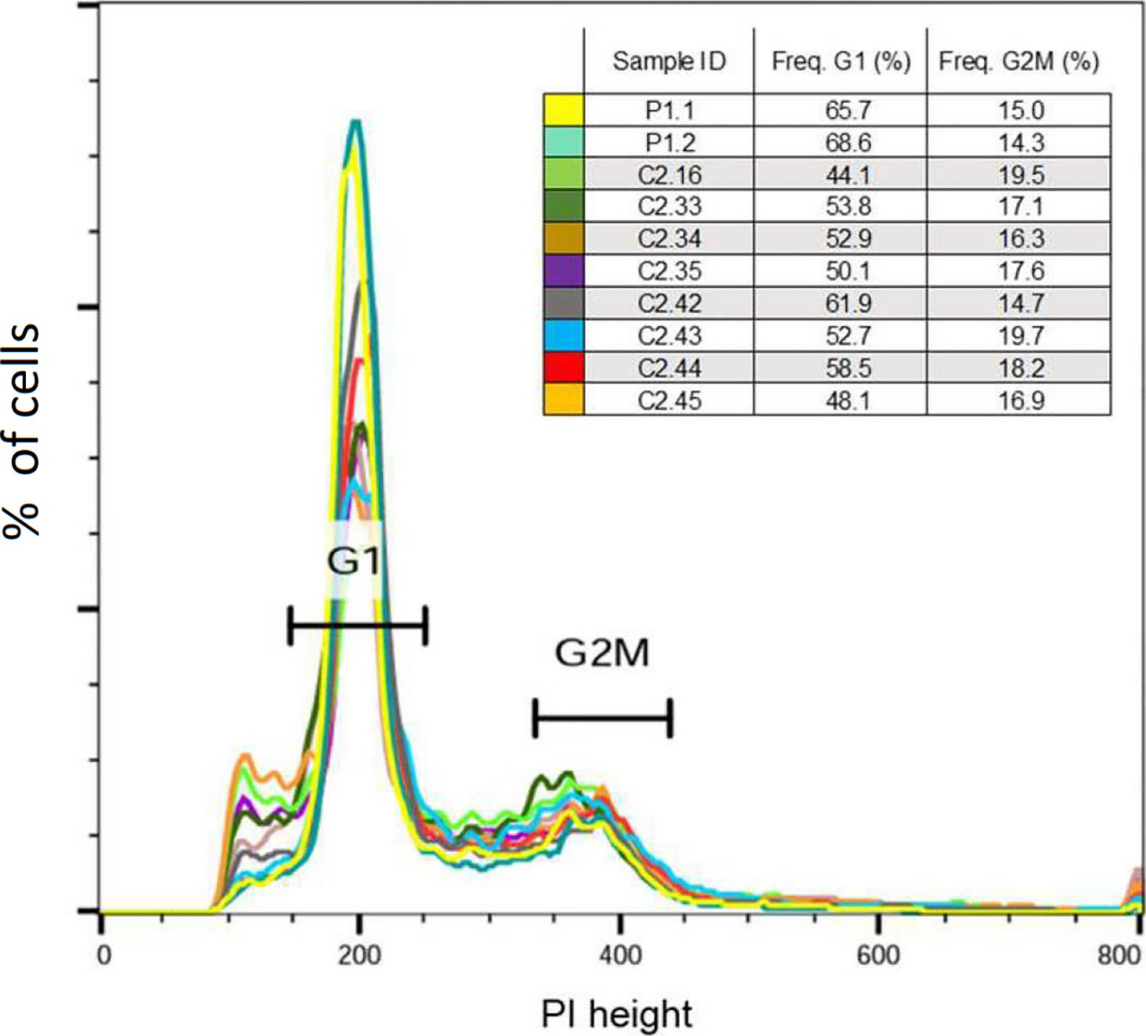
Frequency of G1 and G2/M populations in parental and hybrid clones indicate that hybrids are diploid. PI, Propidium iodide; P1.1 and P1.2, parent GR347.

**Fig 3:**
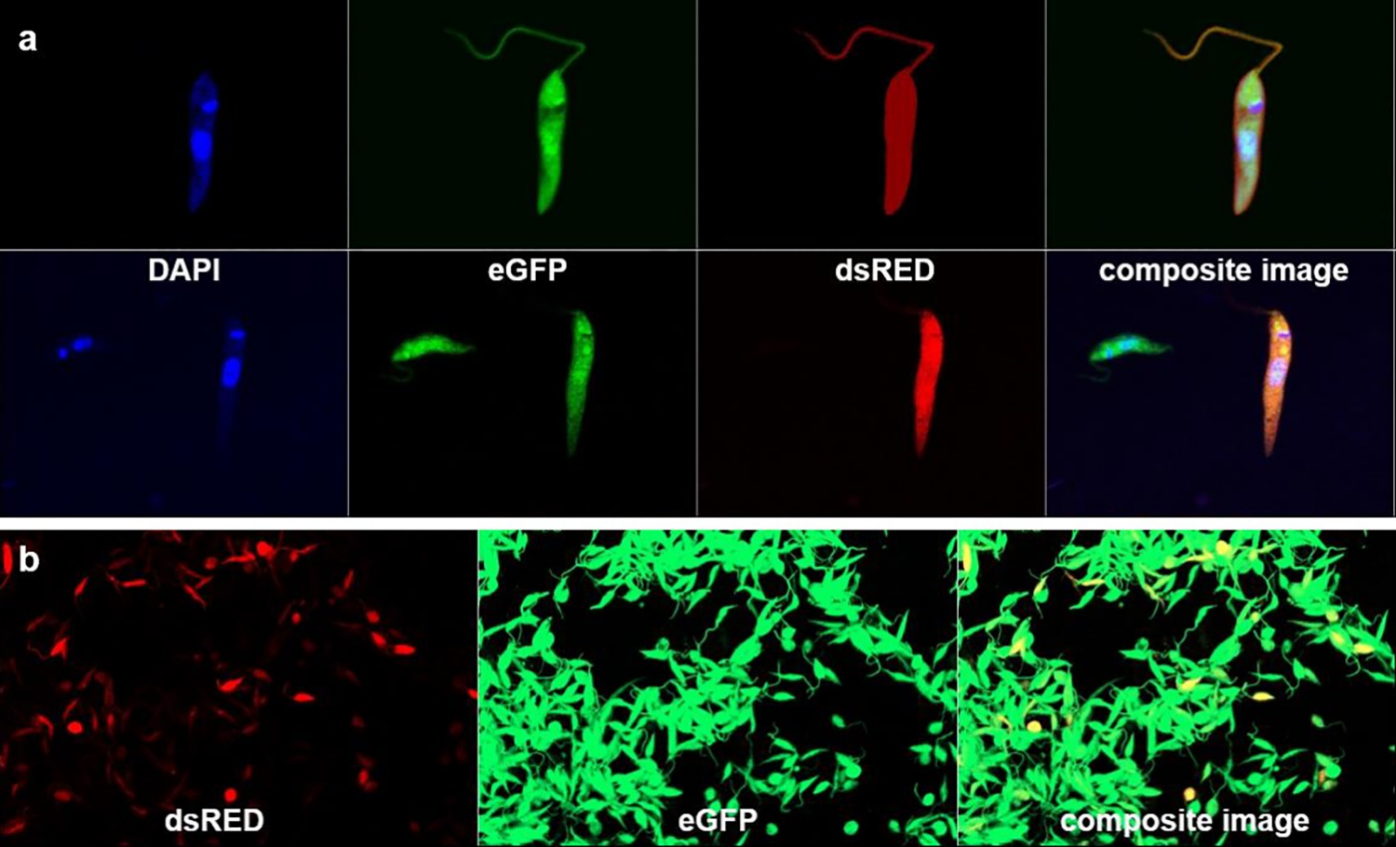
Confocal microscopy of *in vitro* hybrid clones showing: a) DNA (using DAPI) stained, eGFP expressing, dsRED expressing, and dual-expressing yellow hybrid single cells; b) Dual-expressing hybrid cultures, dsRED having a lower level of expression than eGFP.

Of the 19 isolates from dissected sand flies, 41 clonal colonies were obtained, 31 clones from batch 1, 10 from batch 3 and none from batch 2; 14 were derived from *P*. *orientalis* and 27 from *P*. *argentipes*. All the clones were initially pre-screened for green and red fluorescing cells using an epifluorescence microscope (Table 5). Eight clonal isolates, highly expressing both green and red fluorescence, and parental strain GR347 in duplicate, were selected to assess ploidy of the hybrid population (Fig 2). The DNA contents of the parental strain (P1, GR347) and the clonal isolates clearly showed 2n (G1) and 4n (G2/M) peaks. The percentage of cells in the G1 phase for all the samples ranged from 44.1% to 68.6%, while the percentage in the G2/M phase was between 14.3% to 19.7% (Fig 2). Cultures and single clonal hybrids, previously selected using epifluorescence microscopy from batches 1 and 3 and from both *P*. *orientalis* and *P*. *argentipes*, were viewed and hybrids confirmed by confocal microscopy (Fig 3).

**Table 5:** Hybrid clones, derived from *P*. *orientalis* (ORI) and *P*. *argentipes* (ARG), and epifluorescence microscopy. Origins of C1.3, C1.21, C1.23 and C1.25 are uncertain; **+** = presence of fluorescence;— = no fluorescence detected.

Clone ID	Origin	Fluorescence	
		Green	Red
C1.1	ORI9	+	+
C1.2	ARG1	+	+
C1.3	-	+	-
C1.4	ARG1	+	-
C1.5	ARG2	+	-
C1.6	ARG1	+	-
C1.7	ARG3	+	+
C1.8	ARG2	+	-
C1.9	ORI5	+	-
C1.10	ORI10	+	-
C1.11	ORI1	+	-
C1.12	ARG2	+	-
C1.13	ORI10	+	-
C1.14	ORI10	+	-
C1.15	ORI9	+	-
C1.16	ORI5	+	-
C1.17	ORI9	+	-
C1.18	ARG1	+	+
C1.19	ARG1	+	+
C1.20	ORI5	+	-
C1.21	-	+	-
C1.22	ARG2	+	-
C1.23	-	+	-
C1.24	ORI9	+	-
C1.25	-	+	-
C1.26	ARG2	+	-
C1.27	ORI9	+	+
C1.28	ARG3	+	-
C1.29	ORI5	+	-
C1.30	ORI5	+	-
C1.31	ARG3	+	-
C1.32	ARG3/5 P2	+	+
C1.33	ARG3/5 P2	+	+
C1.34	ARG3/5 P2	+	+
C1.35	ARG3/5 P2	+	+
C1.36	ARG3/5 P2	+	+
C1.37	ARG3/5 P2	+	-
C1.38	ARG3/5 P2	+	-
C1.39	ARG3/5 P2	+	-
C1.40	ARG3/5 P2	+	-
C1.41	ARG3/5 P2	+	-

### MLST shows nuclear hybridisation

**Fig 4:**
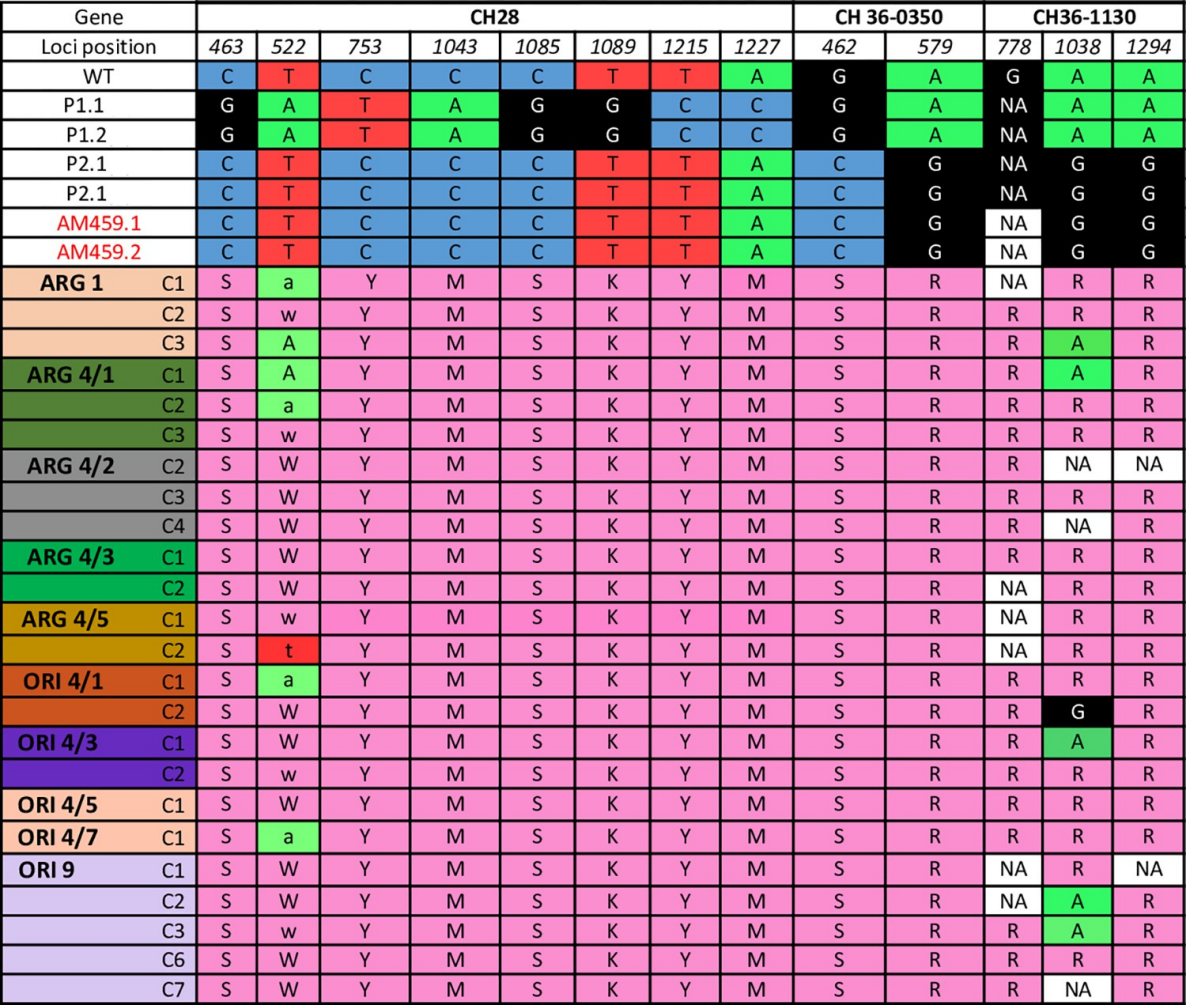
Thirteen relevant SNPs found by MLST analysis of three nuclear markers of parental strains and hybrid clones. WT, Ethiopian *L*. *donovani* wild type. Parental strains P1 (GR347) and P2 (AM459); P1.1 and P1.2 (clones derived from GR347); P2.1 and P2.2 (clones derived from AM459). ARG and ORI denotes hybrid clones derived from *P*. *argentipes* and *P*. *orientalis* respectively. Clones from the same sand fly are indicated by distinct clone ID. SNPs with low level of confidence are denoted with lower cases. NA indicates sequencing data not available; R = G+A; Y = T+C; S = G+C; W = T+A; K = G+T; M = A+C.

**Fig 5:**
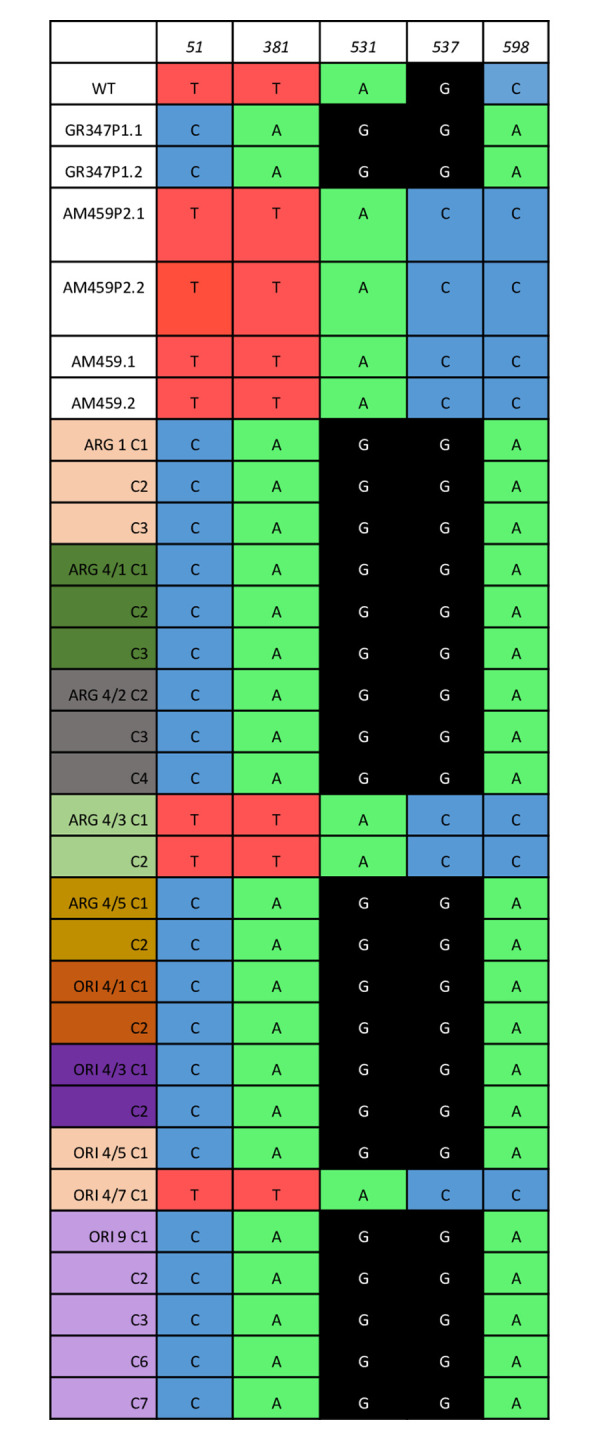
Analysis of cytochrome b SNPs indicates homozygosity of hybrid kinetoplast DNA.

Twenty-four of the hybrid clones were analysed by MLST. From the three nuclear markers, Ch28, Ch36-0350 and Ch36-1130, a total of 13 SNP sites were identified, where parental strains and hybrid clones showed differences, with the hybrid clones possessing allelic heterozygosity at multiple locations (Fig 4). For the cytochrome b kinetoplast gene, whilst the two parental strains possessed distinct genotypes at 5 positions, hybrid clones showed no heterozygosity, possessing only one of the parental genotypes (Fig 5).

### Clonal progeny and chromosomal genomics

**Fig 6:**
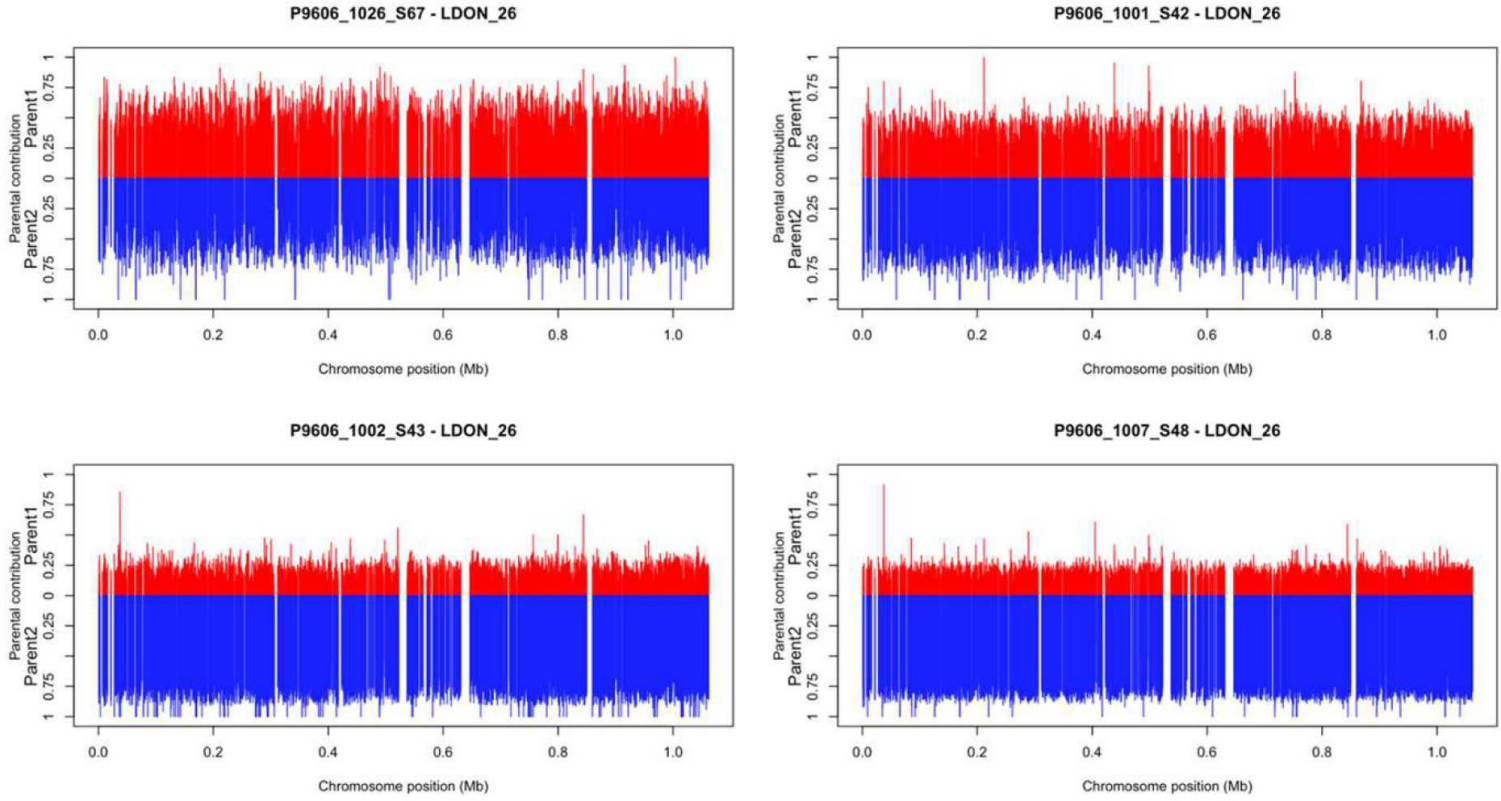
Bottle brush plots show contributions to chromosome 26 by parent 1 (depicted in red) and by parent 2 (depicted in blue) and their diversity in 4 of the hybrid clonal progeny (labelled 1026_S67; 1001_S42; 1002_S43; 1007_S46), with each line representing a SNP position (see text).

Bottle brush plots were used to compare parent 1 (GR347) and parent 2 (AM459) contributions to clonal progeny and to chromosomal diversity. Fig 6 illustrates diversity of chromosome 26 between 4 of the different clonal progeny (labelled 1026_S67; 1001_S42; 1002_S43; 1007_S46). A vertical line is drawn for each SNP position at which parent 1 and parent 2 are homozygous for different alleles. The lines are plotted on the x-axis according to the genomic position on the respective chromosome. The height of the line extending out from the midpoint represents the allele frequency at which both parent 1 (top/ depicted in red) and parent 2 (bottom/ depicted in blue) specific alleles are found in the progeny. The proportional contribution from each parent varies between different sequenced progeny, although at each site a contribution from both parents is almost always observed. As in Figs 6 and S2 plots parent 1 depicted in red and parent 2 depicted in blue to show the allele contributions. However, in S2 Fig contributions are shown for all 36 chromosomes (LDON_1 to LDON_36) of one of the clonal progeny (1026_S67). Hybridisation and allelic diversity are apparent and distributed across all chromosomes of the genome.

### Characterization of HASPB antigen in parents and hybrid progeny

**Fig 7:**
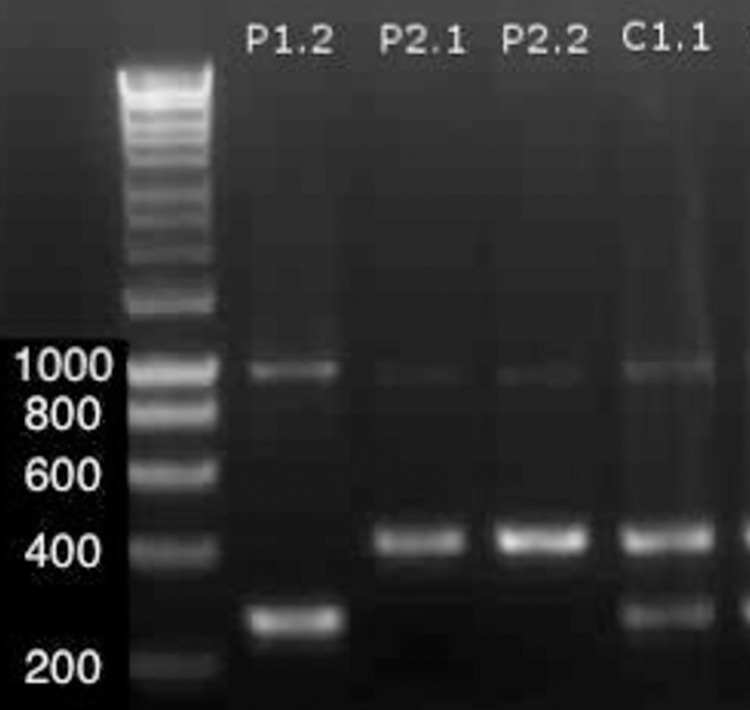
HASPB gene amplification pattern of a representative hybrid clone (C1.1) shows characteristics of both parental strains (P). Sizes (bp) adjacent to DNA ladder.

Amplification of HASPB with primers LdonHASPBFor and LdonHASBPRev revealed two expected fragments of 1064 bp and 260 bp in parent 1, corresponding to repeat coding regions in HASPB1 and HASPB2 respectively. However, the same primers also amplified an unexpected fragment of around 420 bp in parent 2. All 7 hybrid clones (C1.1, C1.2, C1.7, C1.27, C1.32, C1.35, C1.36) had indistinguishable profiles with three amplicons of sizes corresponding with those in parental strains; a representative hybrid is shown in Fig 7. Despite multiple optimization the 1064 bp amplicons could not be sequenced, suggesting divergence in the templates. Sequencing of the 260 bp amplicons yielded the predicted HASPB2 repeated coding region in P1.2 and all 7 hybrid clones, and analysis of the 420 bp band of P2.1 and hybrid clones revealed a HASPB1/HASPB2-like combined composition across their 7 repeat coding regions.

### Genomic diversity of parents and progeny in diagnostic and vaccine targets

NCBI sequences of diagnostic and vaccine candidates were blasted against both parental and progeny genomes. Where function was known, most of the genes had roles either in signalling pathways (both intra- and extracellular) or metabolic pathways. In summary, many of the proteins were diverse between the progeny, including those involved in signalling and metabolism, hypothetical proteins coding for surface antigens, such as enolase, ATP synthase alpha subunit. In contrast, other proteins were highly conserved, such as histones and products of housekeeping genes (Table 6; S2 Fig).

**Table 6:** Missense mutations observed in the *Leishmania* progeny.

Chromosome	Diagnostic/vaccine candidate	GenBank^a^	Amino acid changes (bold) with encoding mutation(s)	PubMed ID
Ldv9_00	Cathepsin L-like protease	XM_003858609.1	92A>92V 1357133C>T; 166A>166V 1357355C>T; 207F>207I 1357477T>A; 298I>298T 1357751T>C; 326L>326K 1357834C>A & 1357835T>A	31660874
Ldv9_00	Sterol 24-c-methyltransferase, putative	XM_024473496.1	281D>281N 1805397G>A	20439959
Ldv9_01	HSP70-like protein	XM_003857831.1	2R>2H 189053G>A; 324I>324S 190019T>G; 589H>589Y 190813C>T; 761V>761G 191330T>G; 830A>830G 191537C>G; 1066R>1066Q 192245G>A	37514945
Ldv9_04	*L*. *infantum* JPCM5 hypothetical protein, conserved in *Leishmania*	XM_001462817.1	30L>30F 48175C>T; 284L>284F 48939G>C	b
Ldv9_04	*L*. *major* Friedlin surface antigen-like protein	XM_883436.1	164P>164T 54845C>A; 178T>178K 54888C>A; 309T>309A 55280A>G; 376P>376T 55481C>A; 482A>482V 55800C>T;	22038252
Ldv9_04	*L*. *major* Friedlin surface antigen-like protein	XM_883437.1	23T>23M 56961C>T; 245R>245K 57627G>A; 687A>687V 58953C>T	b
Ldv9_05	Trypanothione reductase	XM_003858174.1	152E>152D 102972A>C; 213W>213C 103155G>C; 343A>343G 103544C>G	24606556
Ldv9_06	*L*. *major* Friedlin conserved hypothetical protein	XM_001680755.1	21I>21V 128160A>G; 131L>131M 128490C>A	b
Ldv9_06	*L*. *infantum* JPCM5 hypothetical protein, unknown function	XM_001463097.2	103L>103P 171416T>C; 135V>135I 171511G>A; 173V>173A 171626T>C; 474S>474N 172529G>A	b
Ldv9_07	Cysteine peptidase B (CPB)	XM_003858451.1	74G>74E 255384G>A; 110T>110S 255491A>T; 146T>146S 255600C>G; 383C>383R 256310T>C; 1048V>1048A 258306T>C; 1276P>1276A 258989C>G; 1325I>1325M 259138C>G; 1584A>1584V 259914C>T; 1617K>1617Q 260012A>C; 1876A>1876P 260789G>C	15882533
Ldv9_10	GP63, leishmanolysin	XM_003858840.1	16A>16T 206225G>A; 18E>18A 206232A>C; 24A>24P 206249G>C; 33L>33V 206276C>G; 52C>52Y 206334G>A	33910113
Ldv9_12	*L*. *infantum* JPCM5 putative 3’-nucleotidase/nuclease	XM_003392231.1	24L>24M 171669C>A; 177I>177L 172128A>C	24516114
Ldv9_14	Enolase	XM_003859443.1	119G>119A 496397G>C	27047452
Ldv9_17	*L*. *infantum* JPCM5 histone H2B	XM_001464753.1	91Q>91P 610489A>C	21840052
Ldv9_17	*L*. *major* Friedlin conserved hypothetical protein	XM_001682341.1	36A>36V 652603C>T; 72G>72D 652711G>A	b
Ldv9_18	Gamma-glutamylcysteine synthetase, putative	XM_003860132.1	409K>409E 696554A>G	37317296
Ldv9_19	Cysteine peptidase A 4	XM_003860270.1	136R>136C 601818C>T	15882533
Ldv9_21	Methionine aminopeptidase 2, putative	XM_003860565.1	116A>116P 306369G>C; 183A>183T 306570G>A	22502587
Ldv9_23	*L*. *major* Friedlin conserved hypothetical protein	XM_001683294.1	28T>28A 91070A>G	b
Ldv9_24	Proteophosphoglycan (Ppg3)-related protein-like protein	XM_003861080.1	334G>334D 116358G>A; 380H>380N 116495C>A; 845N>845H 117890A>C; 869T>869I 117963C>T	b
Ldv9_24	*L*. *major* Friedlin conserved hypothetical protein	XM_001683617.1	45Q>45R 569308A>G; 107P>107S 569493C>T; 246F>246L 569910T>C; 262P>262S 569958C>T; 279E>279A 570010A>C; 390L>390P 570343T>C; 392C>392W 570350C>G; 404V>404E 570385T>A; 510V>510A 570703T>C; 536R>536C 570780C>T; 538T>538A 570786A>G; 572T>572A 570888A>G; 781G>781E 571516G>A; 913Q>913L 571912A>T & 571913A>T; 1019P>1019S 572229C>T; 1038C>1038R 572286T>C; 1070A>1070V 572383C>T; 1094A>1094T 572454G>A; 1109L>1109F 572499C>T	b
Ldv9_29	ATP-dependent Clp protease subunit, heat shock protein 100	XM_003862505.1	250Y>250F 483037A>T; 260A>260G 483067C>G; 491M>491V 483759A>G; 599G>599D 484084G>A; 640Q>640R 484207A>G; 644A>644H 484218G>C & 484219C>A & 484220G>C; 656H>656Y 484254C>T & 484256C>T; 661Q>661E 484269C>G; 665P>665S 484281C>T	30787142
Ldv9_34	Vacuolar ATP synthase catalytic subunit A, putative	XM_003864457.1	142A>142T 1399110G>A	b
Ldv9_35	hypothetical protein, conserved	XM_003864666.1	288F>288V 481176T>G,	b
Ldv9_35	*L*. *infantum* JPCM5 hypothetical protein, unknown function	XM_001469264.1	21D>21G 1759598A>G; 127A>127T 1759915G>A; 154I>154M 1759998A>G; 181I>181V 1760077A>G; 198E>198V 1760129A>T; 241R>241G 1760257A>G	b
Ldv9_36	*L*. *infantum* JPCM5 putative serine/threonine protein phosphatase 2B catalytic subunit A2.	XM_001469752.1	8C>8G 771942T>G; 83Q>83R 772168A>G	b
Ldv9_36	*L*. *major* Friedlin S-adenosylhomocysteine hydrolase	XM_001686922.1	270I>270S 1467713T>G; 303T>303N 1467812C>A & 1467813G>C; 350S>350T 1467953G>C	24617796

**Footnotes:** PubMed ID refers to publications citing these proteins as diagnostic or vaccine candidates. a) in most cases, the sequence from *L*. *donovani* BPK282 reference genome is given as the highest NCBI BLAST match to the candidate sequence; in some cases, the *L*. *infantum* (JPCM5) or *L*. *major* (Friedlin) reference genomes gave a higher level of similarity, so that is the result is given here; b) these genes have characteristics of diagnostic or vaccine candidates, but have not been described in detail.

## Discussion

Trypanosomatids were originally considered to be entirely or predominantly clonal. With the advent of MLST and comparative genomics, evidence of genetic exchange became more apparent in natural populations of *Leishmania* [25,26]. Initially there were small studies, such as those in Peru [27] that gave convincing evidence of parental *Leishmania* and hybrid progeny. When larger, wide-ranging populations were analysed, encompassing different continents, it became clear that genetic exchange in *Leishmania* was commonplace and abundant [28–32]. Experimental research proved the occurrence of active genetic exchange in sand flies, demonstrated by infecting flies with pairs of transgenic *Leishmania* strains carrying different fluorescence and selective drug resistance markers [33,34].

Transmission of infection was increased among hybrids [35]. The growth in the sand flies was dependent on the structure of *Leishmania* species and the presence of parasite HASPB and SHERP proteins, which could govern whether flies were permissive or resistant to infection [36]. Visualisation of fluorescent hybrids *in situ* was possible by microscopy of sand fly gut contents [37]. Mating competence could be observed in natural and unnatural vectors [4,38]. Extensive genetic diversity is seen in *L*. *donovani* infections [39] and evidence of meiosis sexual recombination in *Leishmania* species [40].

Here, genetic crosses of two parental strains of *L*. *donovani* (GR347 and AM459) were achieved in the two species of sand fly, *P*. *argentipes* and *P*. *orientalis*. Successful crosses of the two parental strains of *Leishmania* in these two sand fly species, which have different geographical origins in India and Africa, confirmed the permissive nature of the vectors. Cloning of hybrid progeny from both *P*. *argentipes* and *P*. *orientalis* produced a series of clonal lines for studies of the nature and diversity of the progeny. The life cycle stages of the hybrids could be clearly visualised within these sand flies, and after cloning, FACS analysis indicated that progeny was predominantly diploid (Fig 2) and diploidy was shown to be consistent with the analyses of sand fly populations, for example by showing genetic profiles of progeny and hybrids among *L*. *donovani* in Ethiopia [7]. In contrast, progeny obtained from crosses under stress *in vitro* appeared to be predominantly tetraploid, and without classical meiosis [9,10].

Multilocus sequence typing targeting three nuclear genes indicated that the progeny were hybrids. In contrast, the kinetoplast cytochrome b target had alternate SNPs derived uniparentally, as has been observed in the kinetoplast genome of other trypanosomatid hybrids [15]. This may be explicable by the complications of communication between kinetoplastid and nuclear genomes.

Genome sequencing confirmed the diversity between cloned hybrid progeny, as exemplified by comparison of a single chromosome between 4 different progeny (Fig 6). Sequencing of all 36 chromosomes of a single progeny genome showed that the hybrid nature of the progeny was consistent across the entire genome, with fluctuations in the allele proportions, as shown in S2 Fig. This extraordinary diversity in the progeny of these genetic crosses was also apparent from the targeted comparative sequence analysis of diagnostic and vaccine candidates, in Table 6. This showed the widespread occurrence of missense mutations across diagnostic and vaccine expression sites of the genomes. In contrast sequence of house-keeping genes was more stable. Aneuploidy in the genomes of *Leishmania* is another manner in which gene expression and function can evolve [9].

Point-of-care diagnostic tests depend on specificity of antigens. Thus, genetic exchange in *Leishmania* can give rise to significant diagnostic challenges. As a simple example, the diversity of HASPB was apparent and of particular interest, because this stage-regulated surface protein, essential for development in sand flies, is considered to be a candidate component of therapeutic *Leishmania* vaccines. To protect efficacy the known diversity of HASPB has been incorporated into a chemotherapeutic vaccine under trial [24].

One approach to diagnostics and vaccine discovery is to apply comparative genomics to analyse the diversity of agents of leishmaniasis or of other infectious agents, collected in endemic regions or that are already present in biological archives. However, this approach can be demanding, ambitious and costly.

As we have seen from our research findings, another powerful approach is to perform genetic crosses in sand flies, analyse the diversity of the diploid progeny and assess the impact of genetic exchange on the diversity of antigen and vaccine candidates. This is an approach that may also be applicable to drug discovery, if potential drug targets are known or can be identified.

Thus, accumulation and conservation of biobanks, should continue to be a crucial part of vaccine, diagnostics and drug discovery. However, herein we have shown the emergence of novel diversity in candidate diagnostic and vaccine coding sequences can occur.

We have also demonstrated that further similar efforts to perform genetic crosses in sand flies can identify strategic high impact targets to guide future control of infectious diseases. Hopefully, the research presented here will encourage future impetus that will continue to benefit from the extraordinary analytical capacity of comparative genomics.

## Supporting information

Genome sequence data described in this article have been deposited in the European Nucleotide Archive under accession numbers ERR12185559 –ERR12185585.

S1 Fig Fluorescent markers. Fluorescent markers inserted into the *Leishmania* 18S ssu rRNA locus by PCR confirmed amplification of a region spanning the internal fluorescent and flanking sequences. Primers 5’ FW AGCACTCTTCAACCGCGAAA; RV GTGTCGAGTGTCTCCTCCTTTT (57°C, 1382 bp amplicon); 3’ FW ATTCGCGATCTCACAGAGGC; RV GGTTCACCTACAGCTACCTTGT (62°C, 1589 bp amplicon).
(PPTX)

S2 Fig Progeny chromosomes. Depicting all 36 chromosomes (LDON_1 to LDON_36) of a single clonal progeny (1026_S67) illustrating hybridization and diversity across the genome.
(PDF)

S1 Table Primers used in amplification of MLST targets for sequencing. T_A_−temperature of annealing; FW–forward primer; RV–reverse primer; bp–base pairs; kDNA–kinetoplast DNA.
(DOCX)

S2 Table European Nucleotide Archive accession numbers of Illumina sequences of hybrid clones. ERR12185567 was from a parental clone.
(DOCX)
