# Supplementary figures and images for: Comparative genomics of Leishmania donovani progeny from genetic crosses in two sand fly species and impact on the diversity of diagnostic and vaccine candidates
Source: PLoS Negl Trop Dis. 2024 Jan 31;18(1):e0011920. doi: 10.1371/journal.pntd.0011920 (PMC10830044; doi:10.1371/journal.pntd.0011920)

## Slide 1
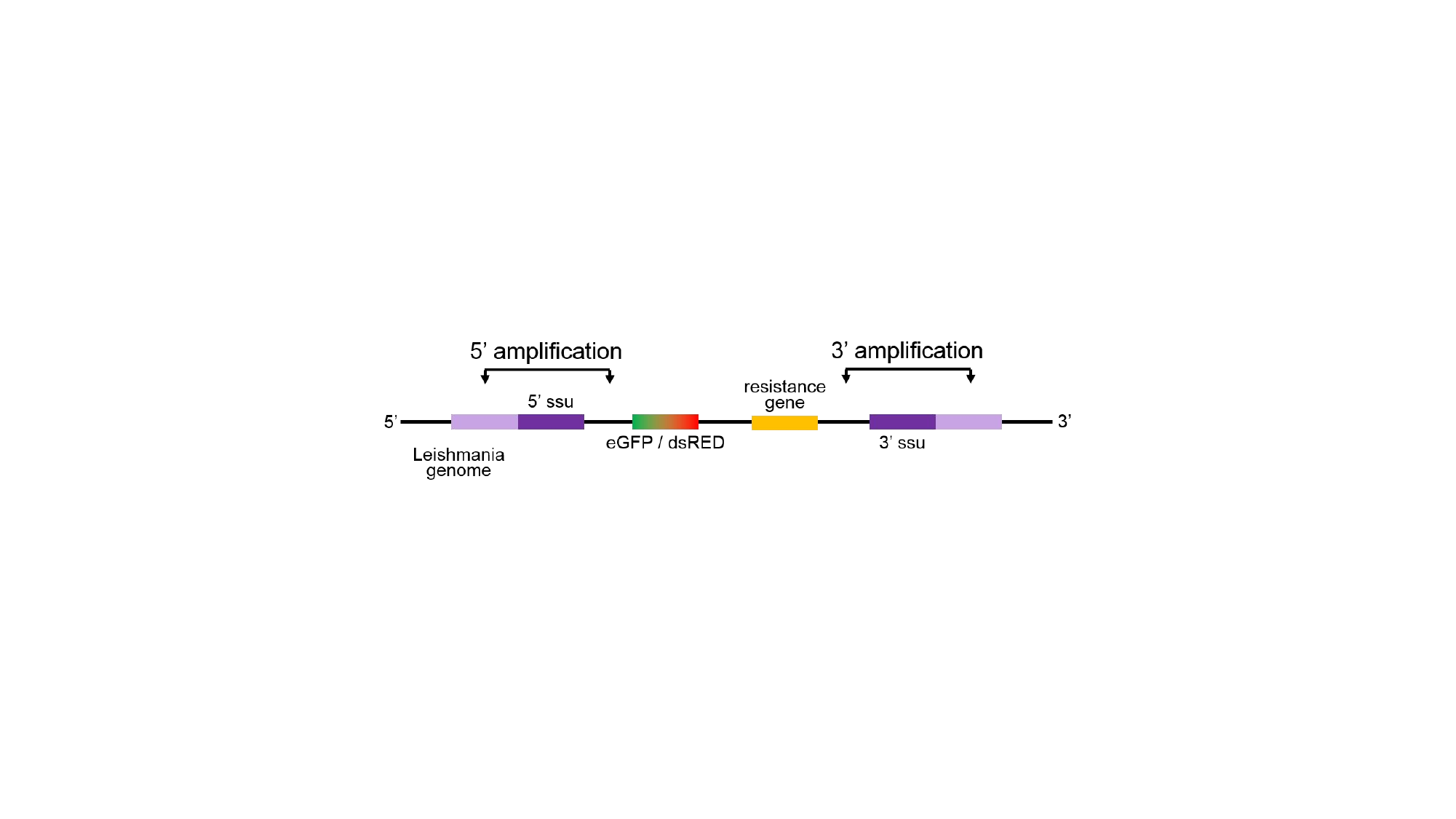

Supplement: S1 Fig — Fluorescent markers inserted into the Leishmania 18S ssu rRNA locus by PCR confirmed amplification of a region spanning the internal fluorescent and flanking sequences. Primers 5’ FW AGCACTCTTCAACCGCGAAA; RV GTGTCGAGTGTCTCCTCCTTTT (57°C, 1382 bp amplicon); 3’ FW ATTCGCGATCTCACAGAGGC; RV GGTTCACCTACAGCTACCTTGT (62°C, 1589 bp amplicon). (PPTX) [file pntd.0011920.s001.pptx]
