# Supplementary material for: Comparative genomics of Leishmania donovani progeny from genetic crosses in two sand fly species and impact on the diversity of diagnostic and vaccine candidates
Source: PLoS Negl Trop Dis. 2024 Jan 31;18(1):e0011920. doi: 10.1371/journal.pntd.0011920 (PMC10830044; doi:10.1371/journal.pntd.0011920)

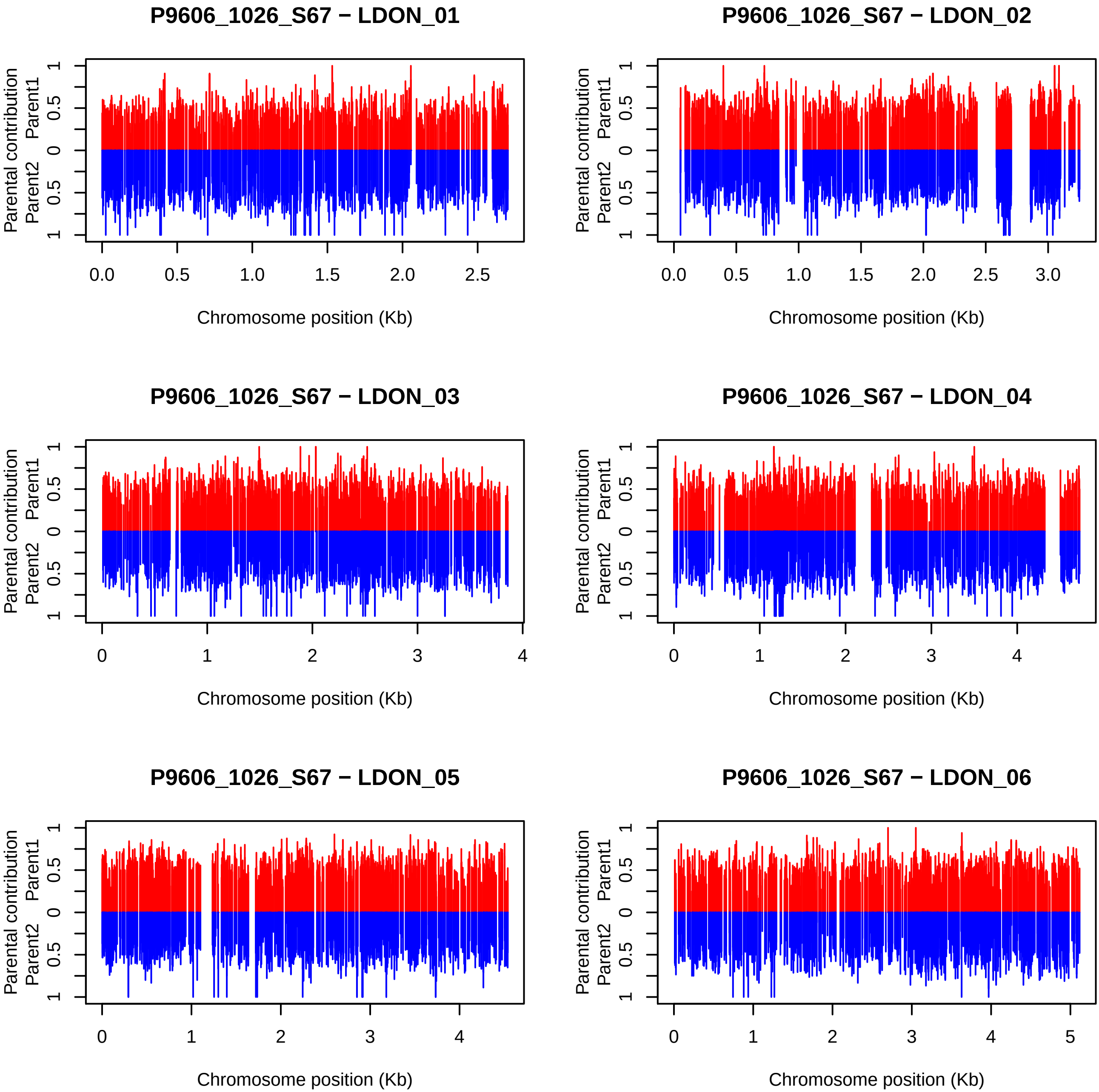
P9606_1026_S67 − LDON_01
P9606_1026_S67 − LDON_02
P9606_1026_S67 − LDON_03
P9606_1026_S67 − LDON_04
P9606_1026_S67 − LDON_05
P9606_1026_S67 − LDON_06
Parental contribution
Parent1
Parent2
1
0.5
0
0.5
1
0.0
0.5
1.0
1.5
2.0
2.5
3.0
0
1
2
3
4
5
Chromosome position (Kb)

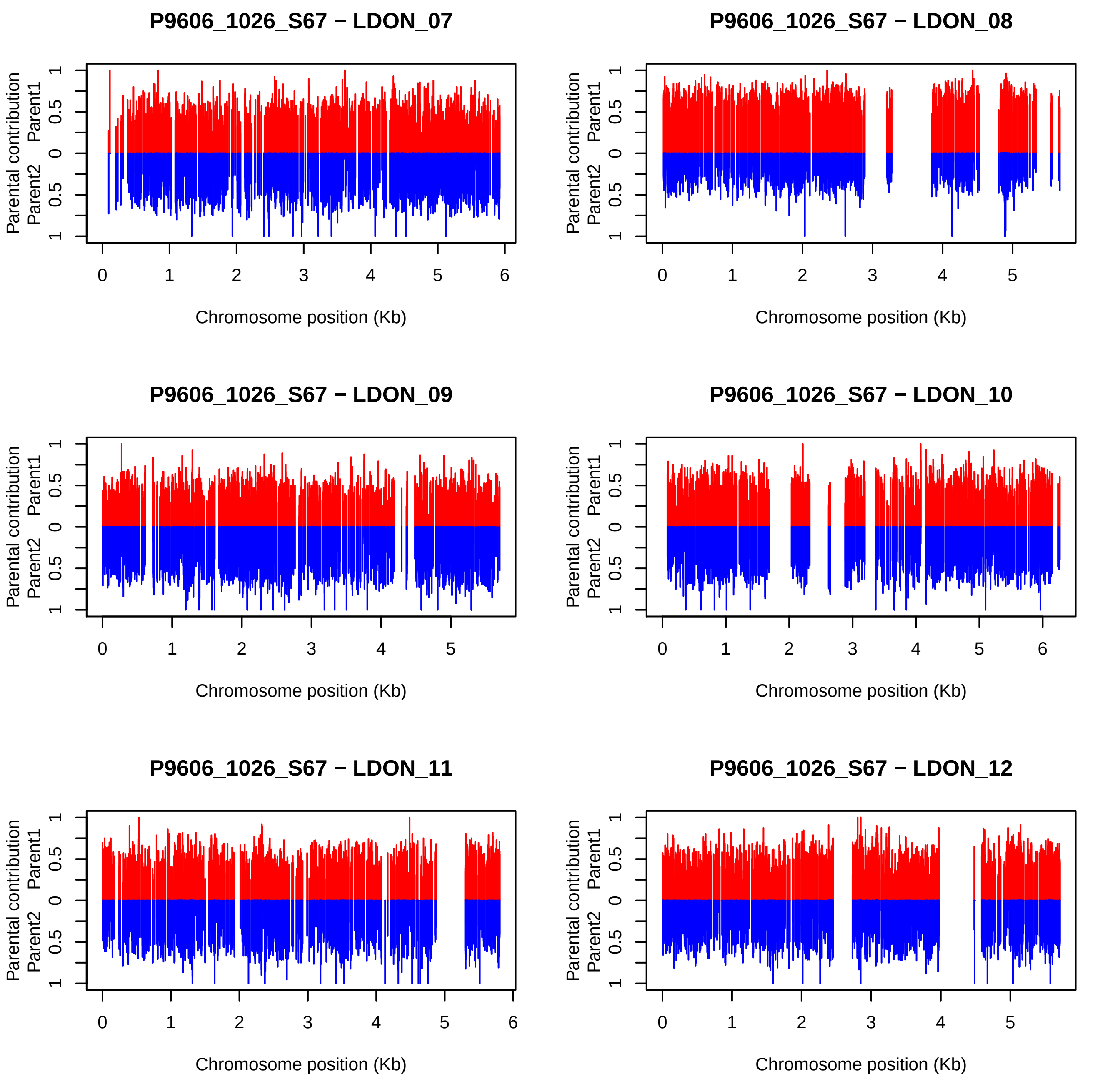
P9606_1026_S67 − LDON_07
P9606_1026_S67 − LDON_08
P9606_1026_S67 − LDON_09
P9606_1026_S67 − LDON_10
P9606_1026_S67 − LDON_11
P9606_1026_S67 − LDON_12
Parental contribution
Parent1
Parent2
1
0.5
0
0.5
1
Chromosome position (Kb)
0
1
2
3
4
5
6

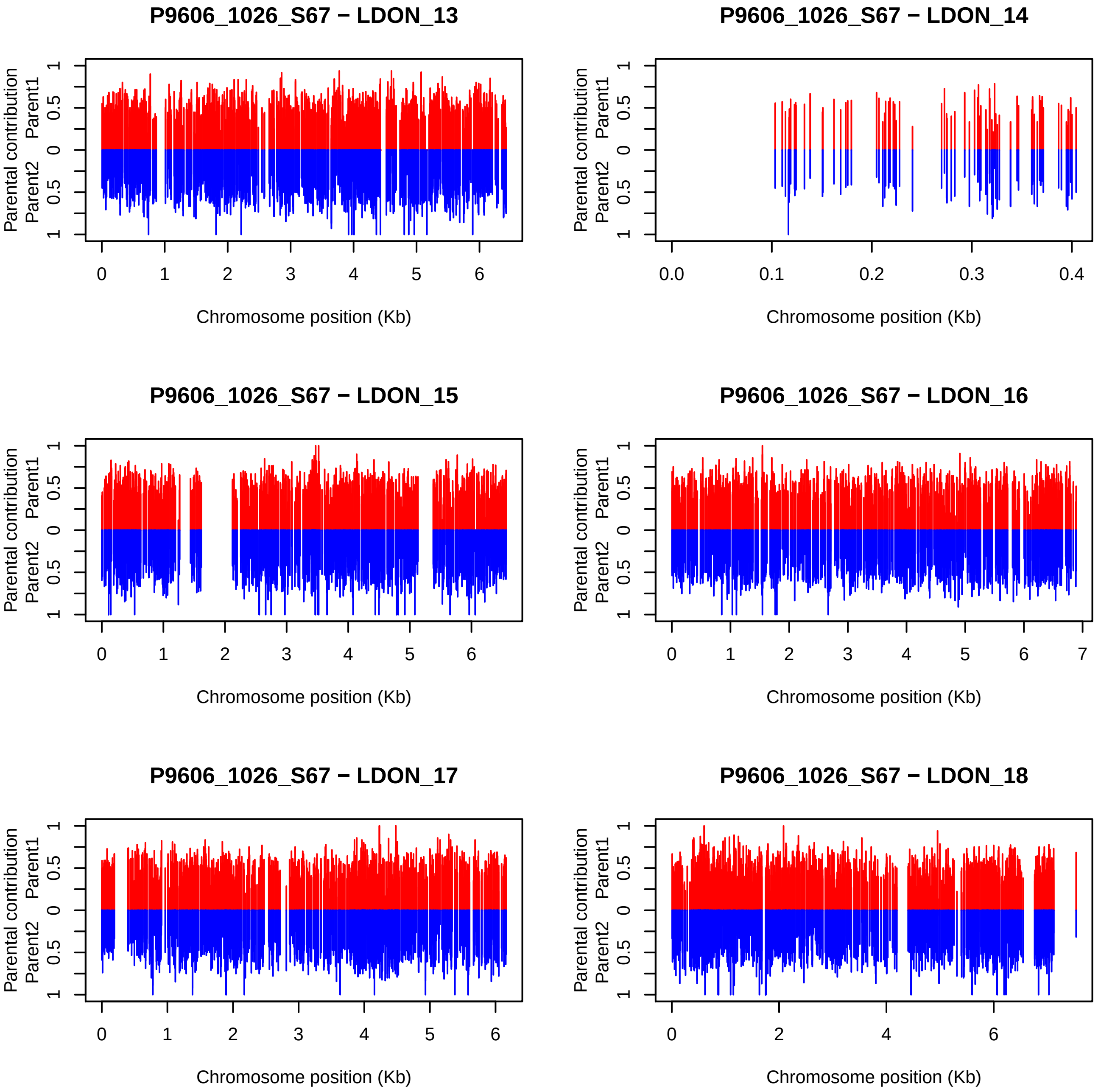

P9606_1026_S67 − LDON_13
P9606_1026_S67 − LDON_14
P9606_1026_S67 − LDON_15
P9606_1026_S67 − LDON_16
P9606_1026_S67 − LDON_17
P9606_1026_S67 − LDON_18
Parental contribution
Parent1
Parent2
1
0.5
0
0.5
1
Chromosome position (Kb)
0
1
2
3
4
5
6
7
0.0
0.1
0.2
0.3
0.4

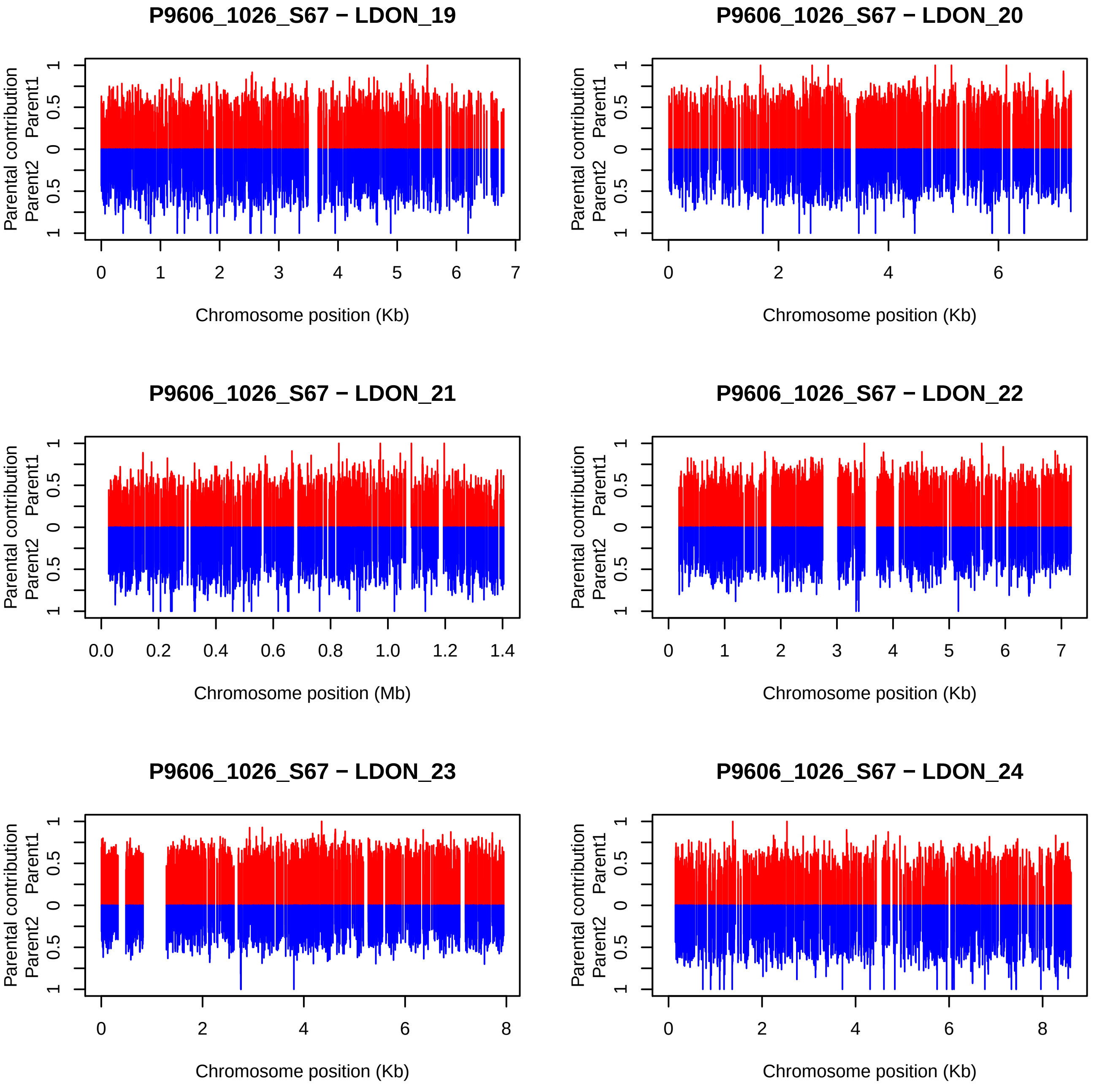
P9606_1026_S67 − LDON_19
Parental contribution
Parent1
Parent2
1
0.5
0
0.5
1
0 1 2 3 4 5 6 7
Chromosome position (Kb)
P9606_1026_S67 − LDON_20
Parental contribution
Parent1
Parent2
0 2 4 6
Chromosome position (Kb)
P9606_1026_S67 − LDON_21
Parental contribution
Parent1
Parent2
0.0 0.2 0.4 0.6 0.8 1.0 1.2 1.4
Chromosome position (Mb)
P9606_1026_S67 − LDON_22
Parental contribution
Parent1
Parent2
0 1 2 3 4 5 6 7
Chromosome position (Kb)
P9606_1026_S67 − LDON_23
Parental contribution
Parent1
Parent2
0 2 4 6 8
Chromosome position (Kb)
P9606_1026_S67 − LDON_24
Parental contribution
Parent1
Parent2
0 2 4 6 8
Chromosome position (Kb)

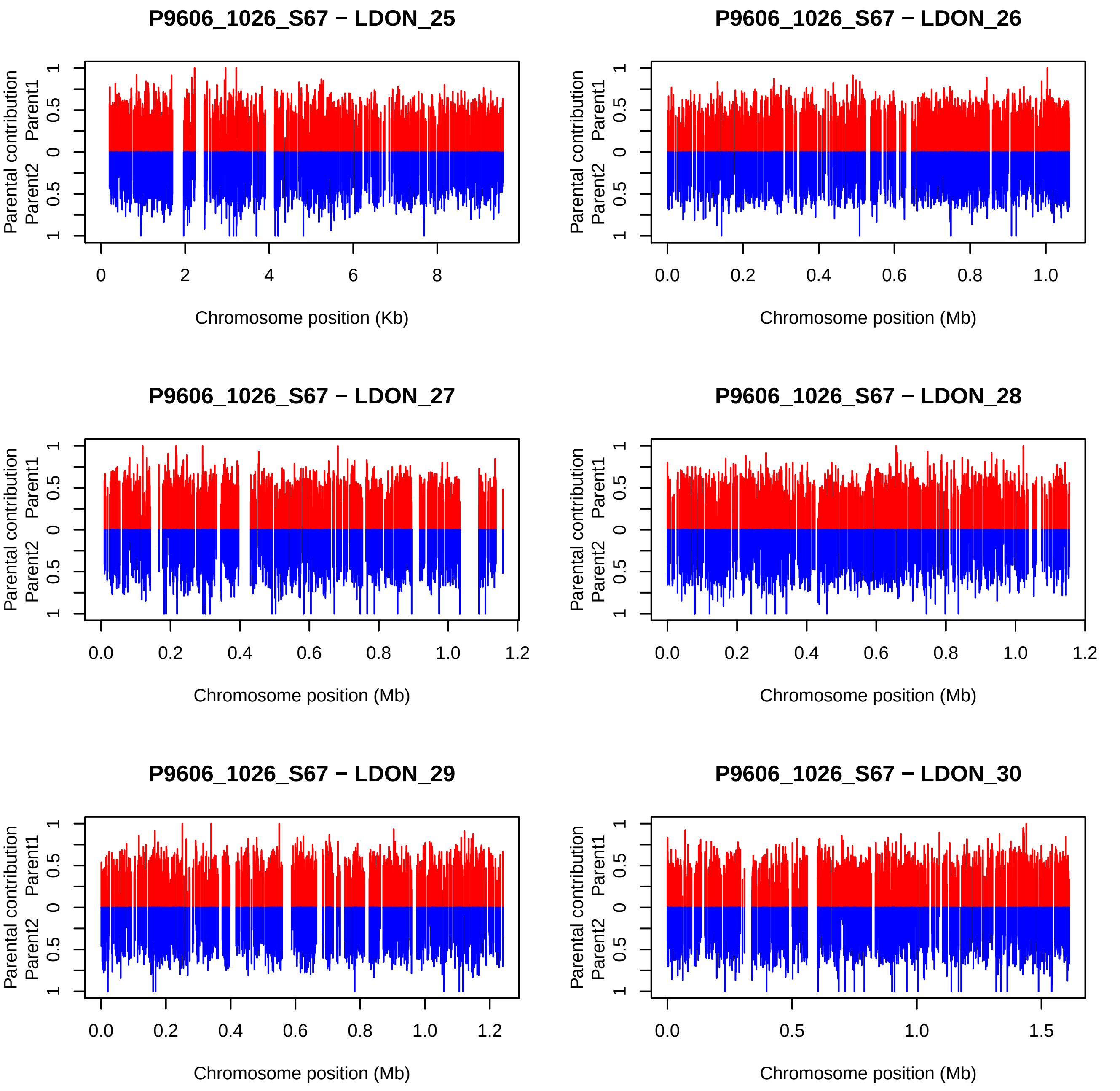
P9606_1026_S67 − LDON_25
Parental contribution
Parent1
Parent2
Chromosome position (Kb)
P9606_1026_S67 − LDON_26
Chromosome position (Mb)
P9606_1026_S67 − LDON_27
Chromosome position (Mb)
P9606_1026_S67 − LDON_28
Chromosome position (Mb)
P9606_1026_S67 − LDON_29
Chromosome position (Mb)
P9606_1026_S67 − LDON_30
Chromosome position (Mb)

P9606_1026_S67 − LDON_31

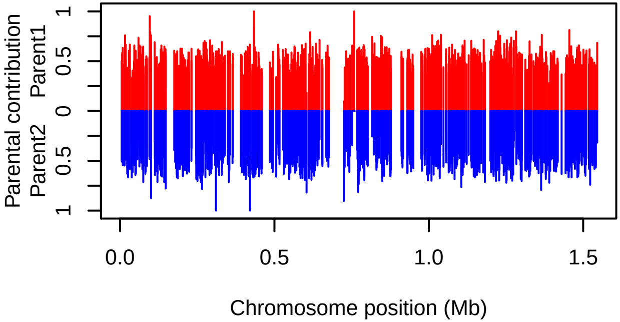

P9606_1026_S67 − LDON_32

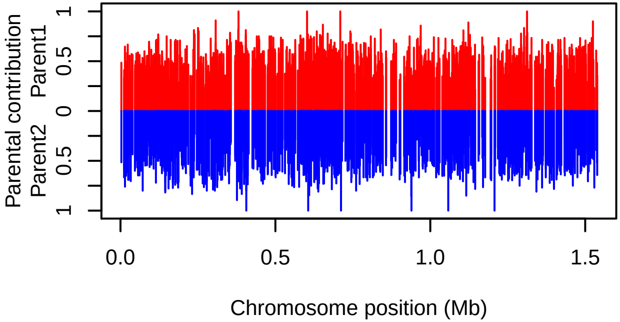

P9606_1026_S67 − LDON_33

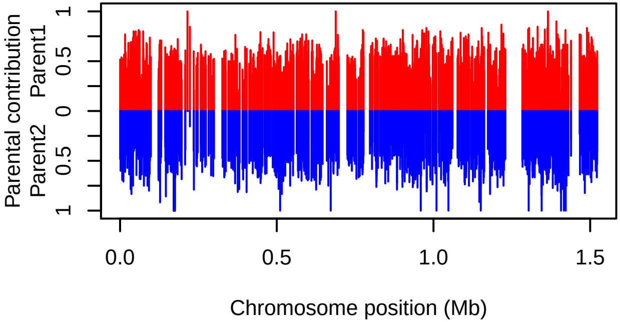

P9606_1026_S67 − LDON_34

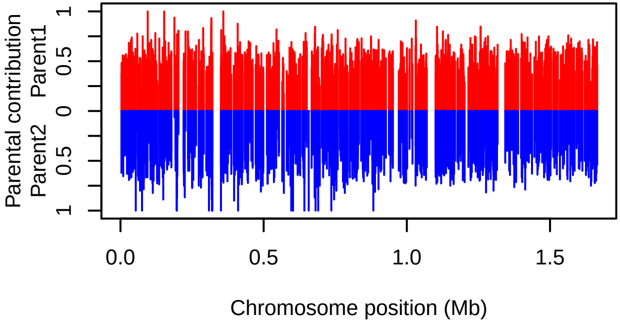

P9606_1026_S67 − LDON_35

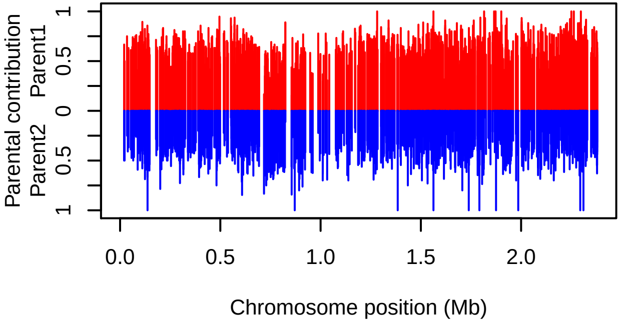

P9606_1026_S67 − LDON_36

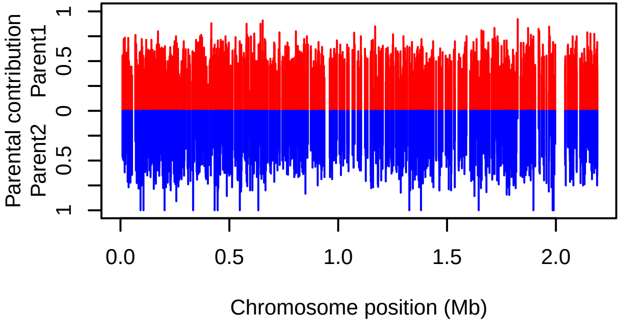

Supplement: S2 Fig — Depicting all 36 chromosomes (LDON_1 to LDON_36) of a single clonal progeny (1026_S67) illustrating hybridization and diversity across the genome. (PDF) [file pntd.0011920.s002.pdf]
