# Supplementary material for: Comparative genomics of Leishmania donovani progeny from genetic crosses in two sand fly species and impact on the diversity of diagnostic and vaccine candidates
Source: PLoS Negl Trop Dis. 2024 Jan 31;18(1):e0011920. doi: 10.1371/journal.pntd.0011920 (PMC10830044; doi:10.1371/journal.pntd.0011920)
Supplement: S1 Table — TA−temperature of annealing; FW–forward primer; RV–reverse primer; bp–base pairs; kDNA–kinetoplast DNA. (DOCX) [file pntd.0011920.s003.docx]

**S1 Table. Primers used in amplification of MLST targets for sequencing.** T_A_ – temperature of annealing; FW – forward primer; RV – reverse primer; bp – base pairs; kDNA – kinetoplast DNA.
