# Supplementary material for: Comparative genomics of Leishmania donovani progeny from genetic crosses in two sand fly species and impact on the diversity of diagnostic and vaccine candidates
Source: PLoS Negl Trop Dis. 2024 Jan 31;18(1):e0011920. doi: 10.1371/journal.pntd.0011920 (PMC10830044; doi:10.1371/journal.pntd.0011920)
Supplement: S2 Table — ERR12185567 was from a parental clone. (DOCX) [file pntd.0011920.s004.docx]

S2 Table. European Nucleotide Archive accession numbers of Illumina sequences of hybrid clones. ERR12185567 was from a parental clone.

| Vector | Illumina label | ENA Accession number |
| --- | --- | --- |
| *P. orientalis* | P9606_1001 | ERR12185571 |
| *P. argentipes* | P9606_1002 | ERR12185576 |
| *P. argentipes* | P9606_1003 | ERR12185572 |
| *P. orientalis* | P9606_1004 | ERR12185574 |
| *P. argentipes* | P9606_1005 | ERR12185570 |
| *P. argentipes* | P9606_1006 | ERR12185578 |
| *P. argentipes* | P9606_1007 | ERR12185580 |
| *P. argentipes* | P9606_1008 | ERR12185559 |
| *P. argentipes* | P9606_1009 | ERR12185581 |
| *P. argentipes* | P9606_1010 | ERR12185579 |
| *P. argentipes* | P9606_1011 | ERR12185569 |
| *P. argentipes* | P9606_1012 | ERR12185582 |
| *P. argentipes* | P9606_1013 | ERR12185583 |
| *P. argentipes* | P9606_1014 | ERR12185584 |
| *P. argentipes* | P9606_1015 | ERR12185573 |
| *P. argentipes* | P9606_1016 | ERR12185564 |
| *P. argentipes* | P9606_1017 | ERR12185560 |
| *P. argentipes* | P9606_1018 | ERR12185566 |
| *P. orientalis* | P9606_1019 | ERR12185562 |
| *P. orientalis* | P9606_1020 | ERR12185585 |
| *P. orientalis* | P9606_1021 | ERR12185568 |
| *P. orientalis* | P9606_1022 | ERR12185577 |
| *P. orientalis* | P9606_1023 | ERR12185563 |
| *P. orientalis* | P9606_1024 | ERR12185561 |
| *P. orientalis* | P9606_1025 | ERR12185565 |
| *P. orientalis* | P9606_1026 | ERR12185575 |
